# Comparative Transcriptomics Analyses Identify DDX43 as a Cellular Regulator Involved in Suppressing HSV-2 Replication

**DOI:** 10.3390/v17101366

**Published:** 2025-10-13

**Authors:** Ranqing Cheng, Yuncheng Li, Yuhao Chen, Mudan Zhang, Qinxue Hu, Yalan Liu

**Affiliations:** 1 Savaid Medical School, University of Chinese Academy of Sciences, Beijing 100049, China; chengranqing22@mails.ucas.ac.cn (R.C.); liyunc97@163.com (Y.L.); micros-7@outlook.com (Y.C.); mudan@wh.iov.cn (M.Z.); 2 State Key Laboratory of Virology and Biosafety, Wuhan Institute of Virology, Chinese Academy of Sciences, Wuhan 430071, China; 3 Hubei Jiangxia Laboratory, Wuhan 430207, China

**Keywords:** transcriptomics, DDX43, HSV-2, IFN-β

## Abstract

HSV-2 is the main pathogen causing genital herpes, and its infection increases the infection and transmission of HIV-1. Currently, there are no vaccines to prevent HSV-2 infection or treatment that can fully cure it. Mining key host factors that regulate HSV-2 replication and elucidating their specific regulatory mechanisms are crucial for understanding virus–host interactions and discovering new antiviral targets. In the current study, we identified DDX43 as a cellular factor involved in the suppression of HSV-2 replication through comparative transcriptomic analyses of HSV-2-infected epithelial cells, followed by experimental validation. Comprehensive transcriptomic profiling revealed distinct host cellular gene expression patterns in HeLa and ARPE-19 cell lines post HSV-2 infection. Subsequent orthogonal partial least-squares discriminant analysis (OPLS-DA) pinpointed DDX43 as one of the principal mediators distinguishing the host response between HSV-2-infected HeLa and ARPE-19 cells. Furthermore, overexpression of DDX43 inhibited HSV-2 replication, whereas knockdown of endogenous DDX43 enhanced HSV-2 replication. Additional experiments revealed that human DDX43 inhibits HSV-2 replication in an interferon-independent manner. This study demonstrates that DDX43 serves as a host regulator against HSV-2 infection, underscoring the power of comparative transcriptomics in identifying novel host proteins that modulate viral replications.

## 1. Introduction

Herpes simplex virus type 2 (HSV-2) is the main pathogen causing genital herpes, primarily infecting the genital epithelium, and can be transmitted to the peripheral nervous system, where it establishes life-long latent infection [1]. Infection with HSV-2 can increase the risk of bacterial vaginosis and cervical cancer. Additionally, patients infected with HSV-2 are at a higher risk of transmitting HIV-1 [2]. Currently there are no vaccines to prevent HSV-2 infection, nor are there drugs that can completely cure it. Therefore, a thorough exploration of key host factors that modulate HSV-2 replication and mechanisms underlying their regulation is crucial for understanding the virus–host interaction network and identifying new antiviral targets.

To date, several host restriction factors that inhibit herpesvirus replication have been identified, with a particular focus on HSV-1. For instance, transmembrane protein with epidermal growth factor (EGF)-like and two follistatin-like domains 1 (TMEFF1) has been characterized as a neuron-specific restriction factor that impairs HSV-1 entry into neurons [3,4]. Peptidylarginine deaminase 3 (PAD3) exhibits a marked inhibitory activity against HSV-1 [5]. Human myxovirus resistance protein B (MxB) restricts replication of herpesviruses by inhibiting the delivery of incoming viral DNA into the nucleus [6,7]. Nevertheless, current understanding of host factors that restrict HSV-2 replication remains limited.

A variety of methodologies have been developed to identify host cell factors that suppress viral replication. With the rapid advancement of high-throughput sequencing technology and bioinformatics, omics approaches have become crucial tools for elucidating virus–host interactions. The transcriptome serves as an information-rich intermediate layer, offering critical insights into disease pathogenesis [8]. Transcriptomics allows for the systematic acquisition of both quantitative and qualitative data on gene transcript abundance and sequence variations across defined spatiotemporal contexts. It also facilitates dynamic tracking gene expression reprogramming in host cells following viral infection, providing molecular evidence to elucidate viral replication mechanisms, host immune response, and more [9,10,11]. Comparative analysis of transcriptome enables the identification of host genes and molecular pathways that play functional roles in viral infection process. In this context, global gene expression changes in cells in response to HSV-2 infection may reflect early and mechanistically significant cellular events.

HSV-2 exhibits broad tropism, predominantly infecting epithelial cells of the skin and mucosa, with a particular preference for genital mucosal cells. In vitro studies have demonstrated that multiple established cell lines are permissive to HSV-2 infection. However, the replication efficiency of HSV-2 varies significantly across different cell lines [12], and the mechanisms underlying these differences has yet to be elucidated. In this study, we found that viral yields and cytopathic effect (CPE) differ significantly between two HSV-2 permissive cell lines, HeLa and ARPE-19. Transcriptome sequencing was utilized to systematically compare and analyze the cellular transcriptomic profiles of these two HSV-2-susceptible cell types across multiple time points post infection, with the aim of identifying potential host cellular factors governing HSV-2 infection. By comparing the transcriptomic profiles across different biological states, such as HSV-2-infected HeLa cells vs. HSV-2-infected ARPE-19 cells, or various times post infection, we identified differentially expressed genes (DEGs) that exhibit both statistical significance and high specificity. As the result, the host protein DDX43 was identified and further validated through experimental assays to explore its anti-viral role in HSV-2 replication.

## 2. Materials and Methods

### 2.1. Cell Lines, Viruses and Antibodies

Human embryonic kidney 293T (HEK 293T) cells, human cervical epithelial cell line HeLa and African green monkey kidney cell line Vero were maintained in Dulbecco’s modified Eagle’s medium (DMEM, Huiying Biotech, Thermo Fisher Scientific, Shanghai, China) supplemented with 10% fetal bovine serum (FBS), 100 Units/mL penicillin and 100 Units/mL streptomycin at 37 °C in 5% CO_2_. Adult Retinal Pigment Epithelial cell line-19 (ARPE-19) was maintained in a 1:1 mixture of Dulbecco’s modified Eagle’s medium and F12 medium (Gibco) supplemented with 10% fetal bovine serum (FBS), 100 Units/mL penicillin and 100 Units/mL streptomycin at 37 °C in 5% CO_2_.

HSV-2 (strain G) was obtained from LGC standards. HSV-2-GFP carrying the complete genome of HSV-2 and green fluorescent protein (GFP) was kindly provided by Dr. Yasushi Kawaguchi, University of Tokyo, Japan. All viruses were propagated in African green monkey kidney cells (Vero). Virus titters were determined by a plaque-forming assay on Vero cells as previously described [12].

Antibody (Ab) against DDX43 was purchased from Proteintech (17591-1-AP and 68454-1-Ig, Wuhan, China). Ab against Flag tag was purchased from Sigma-Aldrich (F1804, Saint Louis, MO, USA). Ab against HSV-2 gD was purchased from Santa Cruz Biotechnology (sc69802, Sata Cruz, CA, USA). Ab against HSV2 gB was purchased from Santa Cruz Biotechnology (sc56987, Sata Cruz, CA, USA). Ab against HSV-2 major capsid protein ICP5 was purchased from Abcam (Ab6508, Cambridge, MA, USA). Abs against β-actin was purchased from Proteintech (66009-1-Ig, Wuhan, China).

### 2.2. RNA Extraction, Transcriptome Sequencing and Quantitative Real-Time PCR (qPCR)

HeLa and ARPE-19 cells were washed with PBS, followed by the addition of HSV-2 (MOI = 1) for an incubation at 37 °C for 1 h. After the removal of viruses, cells were washed three times with PBS and maintained in fresh medium supplemented with 2% FBS. The cells were collected at different time points post infection. Total RNAs were isolated from the cells using the TRIzol^®^ Reagent (15596026CN, Invitrogen, Carlsbad, CA, USA) following the manufacturer’s protocol, then subjected to transcriptome sequencing and qPCR. The transcriptome library construction and RNA sequencing (NovaSeq6000, Illumina, San Diego, CA, USA) were performed by Wuhan Zhiyuan Biotechnology Co., Ltd. (Wuhan, China). The sequencing depth for RNA-Seq is >30X reads/sample, yielding a total data output > 60M reads. cDNA was then synthesized using HiScript II Q RT SuperMix (Vazyme, R223-01, Nanjing, China). The newly synthesized cDNAs were used as templates for the amplification of a highly specific nucleotide region of target genes in qPCR assay. For the detection of HSV-2 genomic DNA, viral DNA was extracted from either HSV-2-infected or mock-infected HeLa and ARPE-19 cells using the QIAamp DNA Blood Mini Kit (51104, QIAGEN, Hilden, Germany). The extracted DNAs were served as templates for the amplification of highly specific nucleotide regions within the ICP0 or gB gene, with GAPDH employed as an internal control. Relative qPCR was performed on a CFX Connect Real-Time PCR System (Bio-Rad, Hercules, CA, USA) using ChamQ SYBR qPCR Master Mix (High ROX Premixed) (Vazyme, Q341-02, Nanjing, China). The final reaction conditions were as follows: 95 °C for 1 min, followed by 40 cycles of 95 °C for 15 s, 60 °C for 15 s, and 72 °C for 45 s. The difference in gene expression was calculated on the basis of 2^−ΔΔCT^ values. The primers used in this study were shown in Appendix A (Table A1).

### 2.3. Transcriptome Data Processing

The human reference genome and annotation files were downloaded from website (http://ftp.ensembl.org/pub/current_gtf/homo_sapiens/Homo_sapiens.GRCh38.104.gtf.gz (accessed on 16 August 2023)). HISAT2 (v2.0.5) software was used to map the RNA-seq reads to the reference genome. After obtaining gene read counts using the GenomicFeatures (v1.30.3) and GenomicAlignments (v1.14.2) packages, differential expression analysis between the 2/4/6/8/16 h time points and the 0 h reference group was performed using the DESeq2 (v1.18.1) package. Differential gene expression analysis was conducted using the DESeq2 package with the following criteria: |log2 fold change (FC)| > 0.25 and adjusted *p*-value < 0.05. All identified differentially expressed genes were subsequently integrated for downstream analytical processing.

### 2.4. Bioinformatic Analysis

The following software versions were employed for transcriptomic data analysis: R (version 4.2.2) and Python (version 3.9). Differentially expressed features were identified from each omic dataset as referred in database searching and data management, as well as RNA sequencing and data processing. Principal component analysis (PCA) was performed with all identified features to explore the largest sources of variation within each omics dataset. Orthogonal Partial Least-Squares Discriminant Analysis (OPLA-DA) was performed using the MetaboAnalyst platform. Time-series data were analyzed using fuzzy c-means clustering with the Mfuzz package (v2.60.0) (parameters: c = 9, m = 1.25). Data visualization was conducted using ggplot2 and Cytoscape (v3.9.1).

### 2.5. Plasmid Construction and Transfection

Human DDX43 gene and Flag-tagged DDX43 were cloned into pcDNA3.1 (+), respectively. The Flag-tagged plasmid expressing IRF-3/5D, RIG-I, MAVS, TBK-1, or cGAS/STING, the internal control plasmid phRL-TK and the reporter plasmids p125-Luc were described in our previous study [13]. All the plasmids were verified by DNA sequencing analysis (Sunny Biotechnology, Shanghai, China). Transfection of plasmids into cells was carried out using ExFect Transfection Reagent (Vazyme, T101-01, Nanjing, China) according to the manufacture’s protocol.

### 2.6. Viral Plaque Assay

HeLa and ARPE-19 cells were infected with HSV-2-GFP (MOI = 0.0001) for 1 h. After the removal of viruses, cells were washed three times with PBS and maintained in fresh medium supplemented with 2% FBS. At 48 hpi, cells were washed twice with PBS, followed by fixation with 4% (*w*/*v*) cold paraformaldehyde for 15 min at room temperature. Nuclei were dyed by DAPI (AR1177, Boster, Wuhan, China). Viral plaques were detected using confocal microscopy (STELLARIS 8 WLL, Leica, Wetzlar, Germany) with a 10× objective.

### 2.7. Western Blotting

The collected cells were lysed in the lysis buffer (1 M Tris-HCl, 150 mM NaCl, 200 mM EDTA.2Na, 1% Triton X-100) supplemented with protease inhibitor cocktail (Roche, 11697498001, Basel, Switzerland). The proteins in supernatants were separated by 12% sodium dodecyl sulfate-polyacrylamide gel electrophoresis (SDS-PAGE) and transferred onto 0.45 µm polyvinylidene difluoride membranes (Millipore, Boston, MA, USA). Nonspecific binding was blocked using 5% non-fat milk in TBST for 1 h at room temperature. The membranes were incubated with appropriate antibodies overnight at 4 °C and then washed three times with TBST, followed by incubation for 1 h with HRP conjugated Goat anti-Rabbit IgG (H + L) (Proteintech, SA00001-2, Wuhan, China) or Goat anti-Mouse IgG (H + L) (Proteintech, SA00001-1, Wuhan, China). After three washes with TBST, the bands were visualized by exposure to ChemiScope System (Clinx, Shanghai, China) following the addition of chemiluminescent substrate (Super ECL Plus, S6009M, US EVERBRIGHT, Shanghai, China; Westernbright ECL, K-12045-D50, Advansta, San Jose, CA, USA). The grayscale values of the Wb bands were analyzed using ImageJ (Version 1.33 h). The relative protein expression levels of DDX43, gB, gD, or ICP5 were quantified through the following procedure: First, the gray value of each target protein band (DDX43, gB, gD, or ICP5) was normalized to the corresponding actin band gray value to obtain a lane-specific ratio. Subsequently, the control sample ratio was designated as 1, and the ratios of all other experimental samples were calibrated by dividing them by the control ratio to yield the final normalized values. The triplicate measurements were then averaged, with the mean value presented beneath the corresponding protein band.

### 2.8. RNA Interference

Three specific shRNAs targeted DDX43 and negative control shRNA (Table A2) were designed and constructed into the pLKO.1 vector according to the manufacturer’s instruction. These plasmids and helper plasmids were cotransfected into 293T cells (0.8 μg pMD2.G, 6.67 μg pSPAX2 and 7.5 μg pLKO.1 per dish) for 48 h to produce lentiviruses. At 2 dpi, the culture medium containing the viruses was harvested, collected and filtered by 0.45 µm membrane, followed by ultracentrifugation at 24,000 rpm for 2 h at 4 °C. The purified lentiviruses were aliquoted and stored at −80 °C. For shRNA knockdown of DDX43, lentiviruses and puromycin (5 μg/mL) were added to HeLa cells pre-seeded in culture dishes for an additional 7 days. Cells were harvested and then subjected to WB assay to measure the expression level of DDX43.

### 2.9. Statistical Analysis

Normality of quantitative data was first analyzed using Graphpad Prism 10.4.1 (GraphPad Software, Inc.). Comparisons among groups in experiments data were performed by a two-way ANOVA followed by Bonferroni’s multiple comparisons test. ns, not significant; * *p* < 0.05, ** *p* < 0.01, *** *p* < 0.001, **** *p* < 0.0001. All analyses were based on at least three biological replicates. All experiments were repeated at least three times. Data are presented as mean values ± SD of three independent experiments.

## 3. Results

### 3.1. Comprehensive Transcriptomic Analysis of Cellular Gene Expression Profiles of HeLa and ARPE-19 Cells in Response to HSV-2 Infection

**Figure 1:**
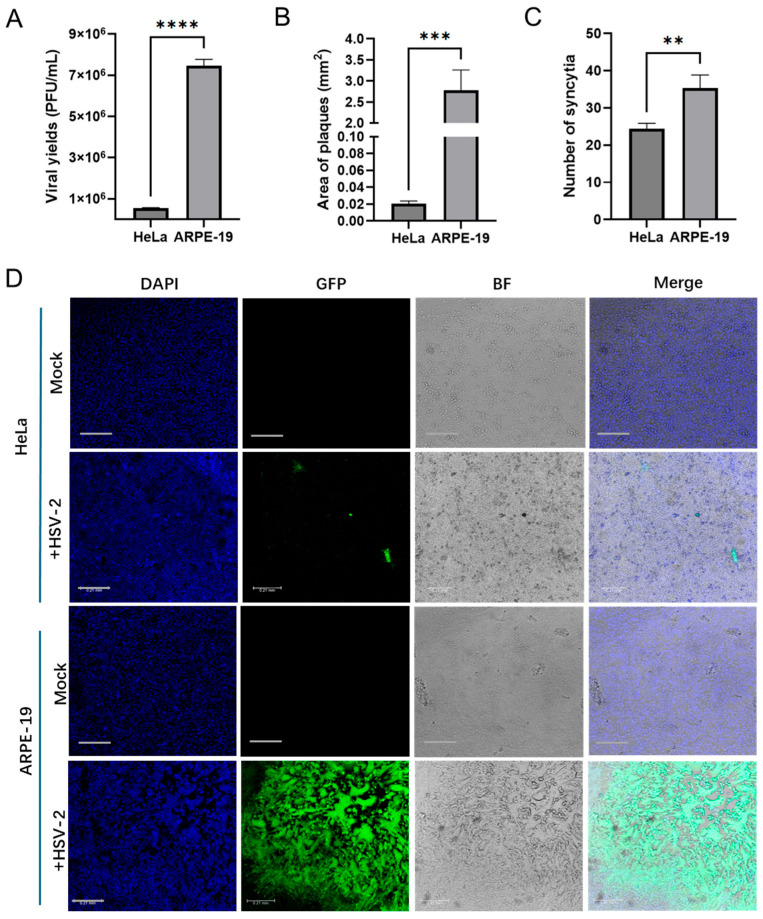
Differential HSV-2 replication in HeLa and ARPE-19 cell lines. (**A**) HeLa or ARPE-19 cells infected with HSV-2 (MOI = 3) for 18 h. Cells and culture medium were collected, and the viral yields were measured by plaque assay. Data shown are mean ± SD of three independent experiments with each condition performed in triplicate. (**** *p* < 0.0001). (**B**–**D**) HeLa or ARPE-19 cells infected with HSV-2-GFP (MOI = 0.0001) for 48 h. The images of plaques were acquired by confocal microscopy. The area of plaques (**B**) and the number of syncytia (**C**) were quantified. The infected cells and syncytium were green. Nuclei were stained with DAPI (blue). Representative confocal images (**D**) from three independent experiments are shown. Scale bars in all panels represent 0.21 mm. For graphs, data shown are mean ± SD of three independent experiments with each condition performed in triplicate (** *p* < 0.01; *** *p* < 0.001; **** *p* < 0.0001).

**Figure 2:**
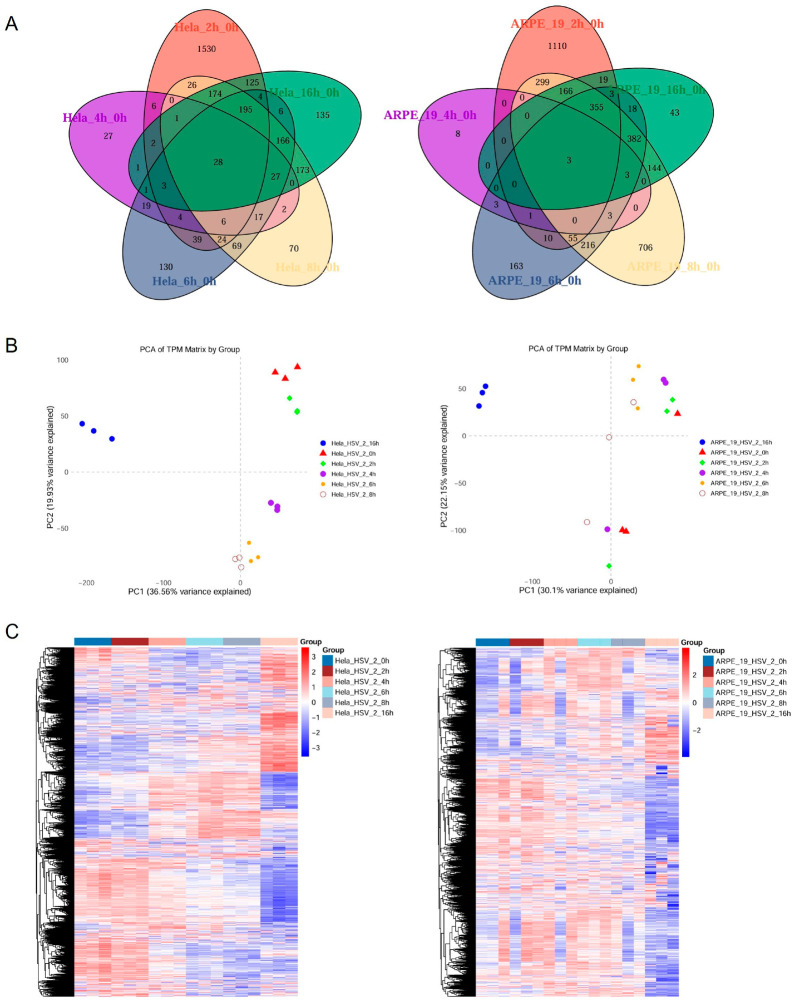
Analysis of the transcriptional expression levels of genes in HSV-2-infected HeLa and ARPE-19 cells. HeLa and ARPE-19 cells infected with HSV-2 (MOI = 1) were collected at different time points (0, 2, 4, 6, 8, 16 h) post infection. The total RNA was extracted for transcriptome sequencing, with the 0 h time point serving as the control group. Comprehensive analysis of the transcriptome sequencing data was conducted, with each group consisting of three biological replicates. (**A**) Venn diagram illustrating the transcriptomic profiles of HeLa and ARPE-19 cells at distinct temporal intervals post HSV-2 infection. A total of 24,135 transcripts were identified in HeLa cells, 28 out of which were consistently present across the samples at five distinct time points. As for ARPE-19 cells, 14,774 transcripts were identified and only 3 were consistently present across the samples at five distinct time points: 2, 4, 6, 8, and 16 h. (**B**) Principle component analysis (PCA) of transcriptome illustrating the transcriptomic profiles of HeLa and ARPE-19 cells at distinct temporal intervals post HSV-2 infection. The control group (0 h) was clearly separated from the other groups (2, 4, 6, 8 and 16 h), indicating significant transcriptomic expression disparities among the samples at various time points post infection, with good reproducibility. (**C**) Clustering heatmap displaying the genes that were upregulated or downregulated in transcriptome samples at different time points post infection.

HeLa and ARPE-19 are commonly used model cell lines in HSV-2 study. Although both cell lines are susceptible to HSV-2, there were significant differences in the yields of progeny viruses and cytopathic effect (CPE) following infection (Figure 1). The yield of progeny viruses in HeLa cells infected with HSV-2 at the same multiplicity of infection (MOI) was significantly lower than that in ARPE-19 cells ((5.5 ± 0.16) × 10^5^ vs. (7.5 ± 0.25) × 10^6^ PFU/mL) (Figure 1A). Additionally, the size of plaques and syncytia in HeLa cells was significantly smaller than that in ARPE-19 cells (0.02 ± 0.002 Vs. 2.78 ± 0.39 mm^2^), while the number of syncytia in HeLa cells was markedly reduced (24 ± 1.25 Vs. 35 ± 2.87 per well) (Figure 1B). To elucidate the mechanisms underlying the differential replication of HSV-2 in various host cell types, we first analyzed the host cellular gene expression profiles in response to HSV-2 infection. HeLa and ARPE-19 cells were collected for transcriptome sequencing at different time points (0, 2, 4, 6, 8, 16 h) post infection, with the 0 h time point serving as the control group. After assessing the quality of raw sequencing data and removing low-quality reads, a thorough analysis of the transcriptome sequencing data was performed. The Venn diagram representing the transcriptomes of HeLa and ARPE-19 cells identified a total of 24,135 transcripts. Among these, 28 transcripts in HeLa cells were consistently present across the samples from five distinct time points: 2, 4, 6, 8, and 16 h. As for ARPE-19 cells, only 3 transcripts were consistently detected across samples from five distinct time points (Figure 2A). Principle component analysis (PCA) revealed that the control group (0 h) was clearly separated from the other groups (2, 4, 6, 8 and 16 h) (Figure 2B), suggesting significant transcriptomic expression disparities among the samples at various time points post infection, with strong reproducibility. The clustering heatmap illustrated the genes that were upregulated or downregulated in the transcriptome samples at different time points post-infection (Figure 2C). These differentially expressed genes are likely to serve as potential regulators influencing HSV-2 replication.

### 3.2. Comparative Transcriptomic Profiling Identifies DDX43 as One of the Principal Mediators Distinguishing HSV-2-Infected HeLa and ARPE-19 Cell Lines

**Figure 3:**
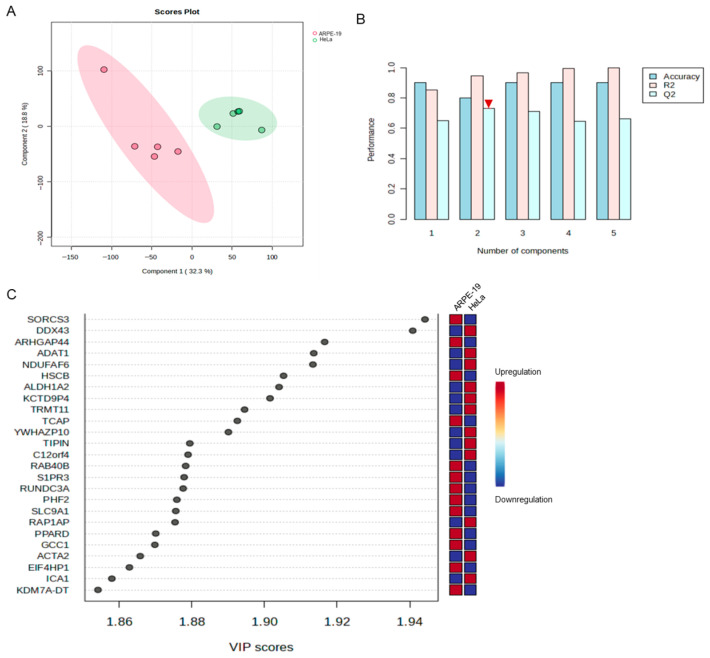
Comparable transcriptomic analyses of the most significant contributors to the transcriptional divergence between HeLa and ARPE-19 cells following HSV-2 infection. An inter-group analysis was carried out on the transcriptomic sequencing data obtained from HSV-2-infected ARPE-19 and HeLa cells, with each group consisting of three biological replicates. (**A**) The PCA scatter plot revealed significant differences in the transcriptional expression levels between ARPE-19 and HeLa cells following HSV-2 infection. 1 is ARPE-19, and 2 is HeLa. (**B**) OPLS-DA was performed on the transcriptome data of the two cell lines using MetaboAnalyst. R2 is the correlation coefficient after PLS-DA analysis, indicating the fitting of the model. Q2 represents the predictive performance of the PLS-DA model. Generally, the values of R2 and Q2 should be relatively close. Among all components, Component #2 shows the closest agreement between R2 and Q2 (indicated by the red inverted triangle). (**C**) The genes with the highest VIP scores, which contributed to the divergence in viral replication levels between the two comparative groups, were identified by using #2 component in (**B**).

An inter-group analysis was subsequently carried out on the transcriptomic sequencing data obtained from ARPE-19 and HeLa cells infected with HSV-2. The PCA scatter plot shows significant differences in the transcriptional expression levels between ARPE-19 and HeLa cells following HSV-2 infection (Figure 3A). Subsequently, orthogonal partial least-squares discriminant analysis (OPLS-DA) was performed on the transcriptome data of the two cell lines using MetaboAnalyst. Based on the OPLS-DA model, the genes with the highest variable importance in the projection (VIP) scores, which contributed to the differences between the two groups, were predicted (Figure 3B,C). As shown in Figure 3B, R2 is the correlation coefficient after PLS-DA analysis, indicating the fitting of the model, that is, the extent to which the established model (component) can represent the real data (generally, when R2 is between 0.7 and 0.8, it indicates that the model has good explanatory power). Q2 represents the predictive performance of the PLS-DA model. In general, a Q2 value greater than 0.5 indicates good predictive ability, and the values of R2 and Q2 should be relatively close. As the #2 component achieved the highest score, it was selected for prediction. Using the #2 component, the top 25 genes with the highest VIP scores were identified, as these genes significantly contribute to the divergence between the two comparative groups (Figure 3C). The higher the VIP value, the more influential the gene is in distinguishing between different groups. Among the top 25 differentially expressed genes, sortilin-related VPS10 domain containing receptor 3 (SORCS3) exhibited the highest score, followed by DEAD-box helicase 43 (DDX43). We focused on genes annotated with antiviral-related functions, such as interferon-stimulated genes (ISGs), and found that neither of these two genes have been previously linked to viral infection. Several members of the DDX family have been demonstrated to play pivotal roles in antiviral defense [14,15,16]. Notably, DDX43 was shown to modulate the interferon (IFN) signaling pathway in *Oreochromis niloticus* [17], suggesting that it may influence HSV-2 infection.

### 3.3. Verification of the Dynamic Expression of DDX43 in Response to HSV-2 Infection

**Figure 4:**
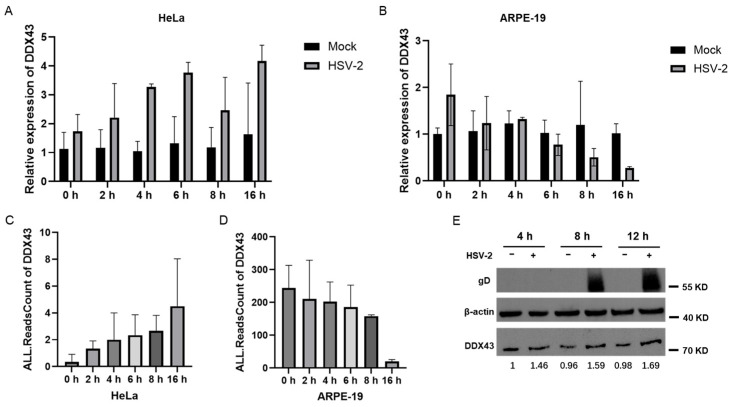
Verification of the dynamic expression of DDX43 in response to HSV-2 infection. HeLa and APRE-19 cells were infected with or without HSV-2, and collected at different time points post infection (0, 2, 4, 6, 8 and 16 hpi), respectively. (**A**) The mRNA level of DDX43 in non-infected and HSV-2-infected HeLa cells were examined using qPCR. (**B**) The mRNA level of DDX43 in non-infected and HSV-2-infected ARPE-19 cells were examined using qPCR. (**C**) The initial transcriptome sequencing results in HeLa cells following HSV-2 infection. (**D**) The initial transcriptome sequencing results in ARPE-19 cells following HSV-2 infection. (**E**) The protein level of DDX43 in HeLa cells was examined by Western blot at different time points following HSV-2 infection. Data shown are mean ± SD of three independent experiments with each condition performed in triplicate. For images, one representative experiment out of three is shown.

DDX43 (also named HAGE), belonging to the DEAD-box helicase subfamily, is a potential prognostic marker in patients with breast cancer [18,19]. It is a dual RNA-DNA helicase, and its KH domain is required for its full unwinding activity [20]. DDX43 regulates RAS protein expression and AKT activation [21], prevents the expression of PML in ABCB5+ malignant melanoma-initiating cells [22], and plays a critical role in the male sex differentiation of channel catfish (*Ictalurus punctatus*) [23]. In mammals, DDX43 plays an important role in the malignant proliferation and immune response of tumor cells [24]. Currently, there is no direct evidence that viral infection affects the expression of DDX43. To verify the accuracy of the aforementioned results from transcriptomics analysis, the dynamics of DDX43 expression in response to HSV-2 infection was assessed. HeLa and APRE-19 cells were infected with or without HSV-2, and collected at different time points post infection (0, 2, 4, 6, 8 and 16 hpi), respectively. The mRNA levels of DDX43 were examined using quantitative real-time PCR (qPCR). As shown in Figure 4A,B, the mRNA level of DDX43 exhibited a progressive increase in HeLa cells, while it gradually declined in ARPE-19 cells during the course of infection, consistent with the initial transcriptome sequencing results (Figure 4C,D). The expression dynamics of genes that are rapidly upregulated in the early stages of infection and remain continuously elevated are more likely to represent key host regulators. The protein level of DDX43 was also examined at different time points following HSV-2 infection, showing that HSV-2 infection resulted in an increase in DDX43 at the protein level in HeLa cells (Figure 4E). The observed concurrent regulation of both mRNA and protein levels of DDX43 during HSV-2 infection suggests that these transcriptional and translational alterations in DDX43 expression are likely mechanistically involved in the pathophysiological processes of HSV-2 infection.

### 3.4. Overexpression of DDX43 Inhibits HSV-2 Replication

**Figure 5:**
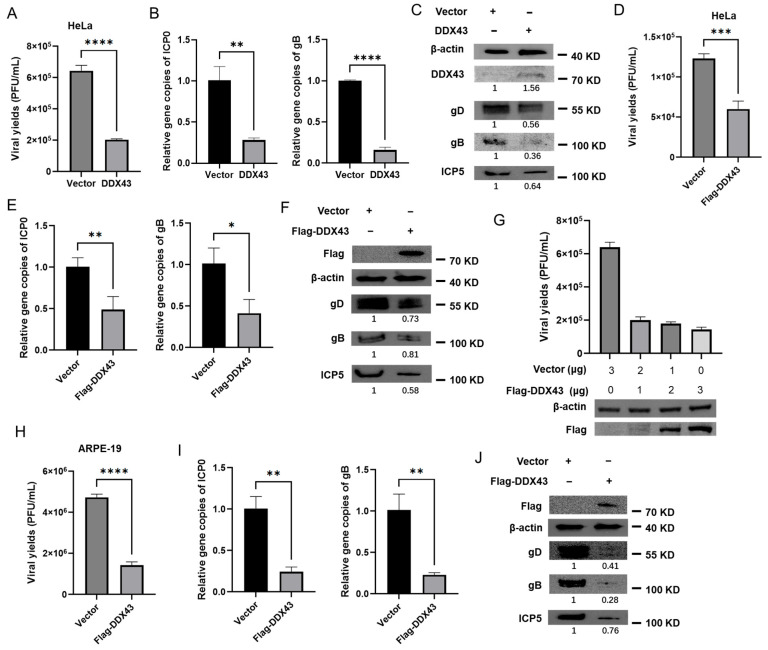
Overexpression of DDX43 inhibits HSV-2 replication. (**A**–**C**) HeLa cells transfected with pcDNA3.1 (+) empty vector or plasmid expressing DDX43. At 24 h post transfection, cells were infected with HSV-2 (MOI = 3) for 18 h, and the cell and culture medium were collected. The viral yields were measured by plaque assay (**A**). The HSV-2 genome was detected by qPCR using ICP0 and gB primers (**B**). The protein expression levels of DDX43, HSV-2 gD, gB, and ICP5 were measured by Western blot (**C**). (**D**–**F**) HeLa cells transfected with pcDNA3.1 (+) empty vector or plasmid expressing Flag-DDX43. At 24 h post transfection, cells were infected with HSV-2 (MOI = 3) for 18 h, and the cell and culture medium were collected. The viral yields were measured by plaque assay (**D**). The HSV-2 genome was detected by qPCR using ICP0 and gB primers (**E**). The protein expression levels of DDX43, HSV-2 gD, gB, and ICP5 were measured by Western blot (**F**). (**G**) HeLa cells were transfected with varying concentrations of plasmid encoding Flag-DDX43, followed by infection with HSV-2. At 18 hpi, the viral yields were detected by plaque assay and the protein expression level of DDX43 was measured by Western blot. (**H**–**J**) ARPE-19 cells transfected with pcDNA3.1 (+) empty vector or plasmid expressing Flag-DDX43. At 24 h post transfection, cells were infected with HSV-2 (MOI = 3) for 18 h, and the cell and culture medium were collected. The viral yields were measured by plaque assay (**H**). The HSV-2 genome was detected by qPCR using ICP0 and gB primers (**I**). The protein expression levels of DDX43, HSV-2 gD, gB, and ICP5 were measured by Western blot (**J**). For graphs, data shown are mean ± SD of three independent experiments with each condition performed in triplicate (* *p* < 0.05; ** *p* < 0.01; *** *p* < 0.001; **** *p* < 0.0001). For images, one representative experiment out of three is shown.

We next sought to determine whether the continuously increasing expression of DDX43 in HSV-2-infected HeLa cells contributes to the lower viral yield. To investigate whether DDX43 functions as a cellular regulator influencing HSV-2 proliferation, HeLa cells were transfected with an empty vector or a plasmid encoding DDX43, followed by HSV-2 infection. At 18 hpi, the cells were collected for qPCR, western blotting, and plaque assays, respectively. As shown in Figure 5, overexpression of DDX43 reduced the viral yields ((6.4 ± 0.29) × 10^5^ vs. (2.0 ± 0.05) × 10^5^ PFU/mL) (Figure 5A), significantly decreased the relative abundance of HSV-2 genome (as indicated by the gene copies of immediately early protein ICP0 and envelope glycoprotein B (gB)) relative to internal reference protein GAPDH (Figure 5B), and downregulated the protein expression levels of major capsid protein ICP5, gB and envelope glycoprotein D (gD) (Figure 5C). To further substantiate the occurrence of DDX43 overexpression, a tagged DDX43 construct was employed to distinguish it from endogenous levels, and the overexpression experiments were repeated. As shown in Figure 5D–F, the results obtained with Flag-DDX43 overexpression were consistent with the previous findings. To explore the correlation between DDX43 expression and HSV-2 proliferation, HeLa cells were transfected with varying concentrations of empty vector and a plasmid encoding Flag-DDX43, followed by HSV-2 infection. As shown in Figure 5G, overexpression of Flag-DDX43 significantly reduced the yield of HSV-2 ((6.7 ± 0.65) × 10^5^ vs. (2.0 ± 0.16) × 10^5^ vs. (1.8 ± 0.08) × 10^5^ vs. (1.4 ± 0.12) × 10^5^ PFU/mL). Moreover, the extent of viral reduction is positively correlated with the transfection dosage of Flag-DDX43. The antiviral activity of DDX43 was further confirmed in ARPE-19 cells. Briefly, ARPE-19 cells were transfected with either an empty vector or a Flag-DDX43-expressing plasmid, followed by HSV-2 infection. At 18 hpi, samples were collected for western blotting and plaque assays, respectively. The results are consistent with those on HeLa cells. Overexpression of Flag-DDX43 reduced the viral yields ((4.7 ± 0.12) × 10^6^ vs. (1.4 ± 0.12) × 10^6^ PFU/mL) (Figure 5H), decreased the relative abundance of HSV-2 genome (Figure 5I) and downregulated the protein expression levels of ICP5, gB and gD (Figure 5J). These data suggest that DDX43 is a cellular regulator that suppresses HSV-2 replication.

### 3.5. Knockdown of Endogenous DDX43 Enhances HSV-2 Replication

**Figure 6:**
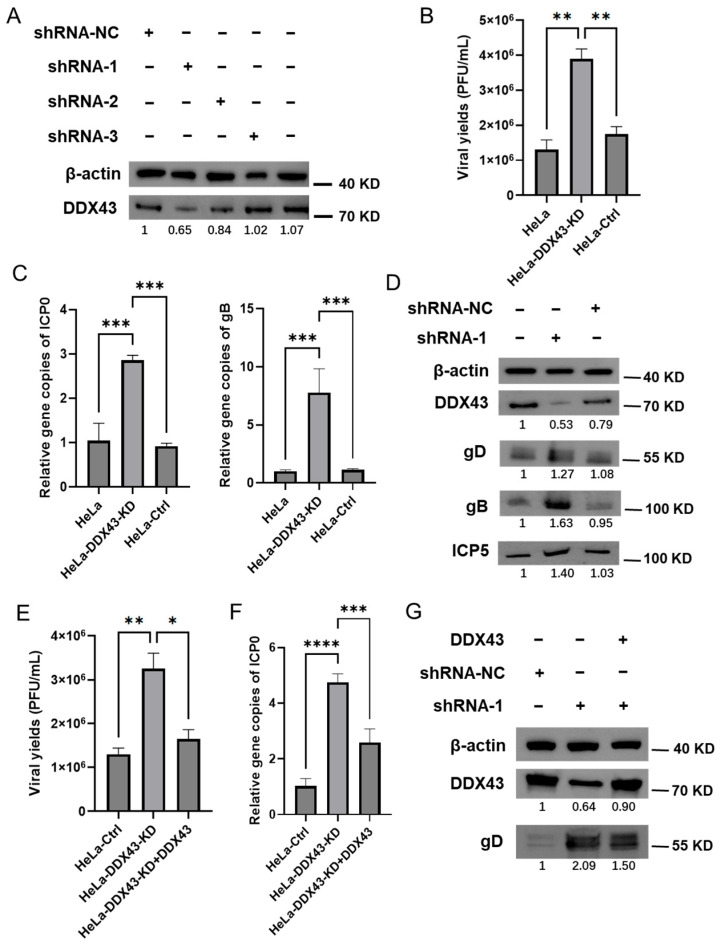
Knockdown of endogenous DDX43 enhances HSV-2 replication. (**A**) Three pairs of shRNAs specifically targeting DDX43 (shRNA-1, shRNA-2, and shRNA-3) and negative control shRNA (shRNA NC) were constructed into pLKO.1 vector and then transfected into 293T cells. The harvested lentiviruses were used to infect HeLa cells to construct a DDX43 knockdown cell line. DDX43 knockdown cells were harvested and then subjected to Western Blot assay to measure the protein expression level of DDX43. (**B**) The viral yields were measured by plaque assay. (**C**) The HSV-2 genome was detected by qPCR using ICP0 and gB primers. (**D**) The protein expression levels of DDX43, HSV-2 envelope glycoprotein gD/gB, and ICP5 were measured by Western Blot. (**E**–**G**) DDX43 expressing plasmid was transfected into HeLa-DDX43-KD (designated as HeLa-DDX43-KD + DDX43). At 24 h post transfection, HSV-2 was used to infect HeLa-Ctrl, HeLa-DDX43-KD and HeLa-DDX43-KD + DDX43 (MOI = 3). At 18 hpi, the viral yields were measured by plaque assay (**E**), the HSV-2 genomes were detected by qPCR using ICP0 primers (**F**), and the protein expression levels of HSV-2 gD and DDX43 were measured by Western Blot (**G**). For graphs, data shown are mean ± SD of three independent experiments with each condition performed in triplicate (* *p* < 0. 05; ** *p* < 0.01; *** *p* < 0.001; **** *p* < 0.0001). For images, one representative experiment out of three is shown.

To further validate the function of DDX43 in HSV-2 infection, retroviral vectors expressing DDX43 shRNA or control shRNA were used. Western blot showed that, at 48 h post transfection, both #1 and #2 DDX43 shRNAs effectively downregulated the expression of DDX43, with #1 DDX43 shRNA demonstrating superior knockdown efficiency compared to #2 shRNA (Figure 6A). Subsequently, #1 DDX43 shRNA was used in the subsequent experiments. HeLa cells transduced with DDX43 shRNA (designated as HeLa-DDX43-KD) or control shRNA (designated as HeLa-Ctrl) were infected with HSV-2. As shown in Figure 6, knockdown of endogenous DDX43 enhanced viral production (Figure 6B) and increased the relative abundance of HSV-2 genome (as indicated by the gene copies of ICP0 and gB) (Figure 6C). Furthermore, HeLa cells transfected with DDX43 shRNA exhibited markedly higher protein expression levels of HSV-2 gD, gB, and ICP5 compared to cells treated with control shRNA, or wild-type HeLa cells (Figure 6D). The anti-HSV-2 effect of DDX43 was further confirmed by reintroducing DDX43-expressing plasmids into HeLa-DDX43-KD. After replenishing DDX43 in the DDX43-knockdown cells, the inhibition of viral replication was restored. The viral yields (Figure 6E), the relative abundance of HSV-2 genome (as indicated by the gene copies of ICP0) (Figure 6F), and the protein expression levels of HSV-2 gD (Figure 6G) in the HeLa-DDX43-KD with DDX43 replenishment were comparable to those in HeLa-Ctrl, but remained lower than those in HeLa-DDX43-KD. These findings further confirmed that DDX43 serves as an intrinsic cellular regulator that suppresses HSV-2 replication.

### 3.6. Human DDX43 Inhibits Viral Replication in an Interferon-Independent Manner

**Figure 7:**
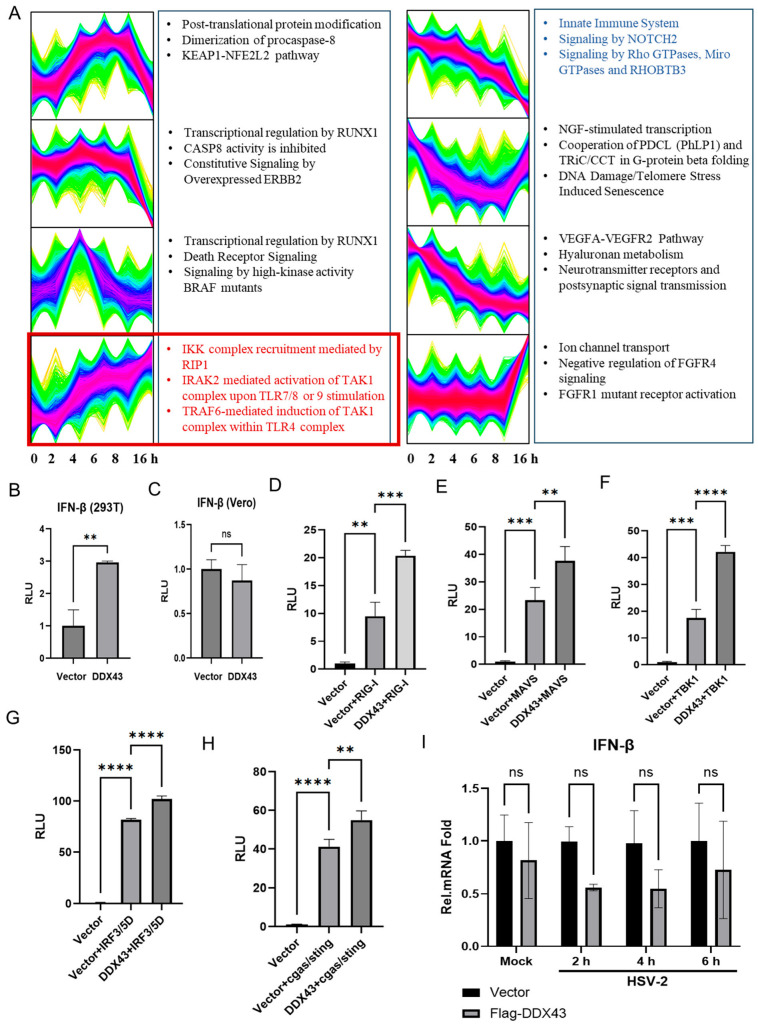
DDX43 inhibits viral replication in an interferon-independent manner. (**A**) Temporal analysis and associated pathways of transcriptome. Using fuzzy c-means clustering, 44,803 mRNAs were clustered into 8 discrete expression clusters to illustrate the relative expression changes in transcriptomes in HeLa cells infected with HSV-2. The top 3 pathways enriched in each cluster in the DAVID database were listed next to the clusters. The key pathways highlighted in red are continuously upregulated during HSV-2 infection, while those highlighted in blue are continuously downregulated. (**B**,**C**) HEK 293T and Vero cells were cotransfected with 440 ng of pcDNA3.1 (+) or plasmid expressing DDX43, 50 ng IFN-β-Luc promoter reporter plasmid p125-Luc and 10 ng internal control plasmid phRL-TK. At 24 h post transfection, cells were lysed, and reporter activities were determined by dual luciferase report (DLR) assay. RLU, Relative Luciferase Unit. (**D**–**H**) HEK 293T cells were co-transfected with either the empty vector pcDNA3.1 (+) or DDX43-expressing plasmid, along with plasmids expressing the components of IFN signaling pathway (RIG-I (**D**), MAVS (**E**), TBK-1 (**F**), IRF-3/5D (**G**) and cGAS/STING (**H**)), reporter plasmid p125-Luc, and internal control plasmid phRL-TK. At 24 h post transfection, cells were lysed for DLR assay. (**I**) HEK 293T cells were co-transfected with either the empty vector pcDNA3.1 (+) or Flag-DDX43-expressing plasmid. At 24 h post transfection, cells were infected with HSV-2 (MOI = 1) for 0, 2, 4, 6 h. Cells were collected and the mRNA expression level of IFN-β was detected by qPCR. For graphs, data shown are mean ± SD of three independent experiments with each condition performed in triplicate (ns, not significant; ** *p* < 0.01; *** *p* < 0.001; **** *p* < 0.0001).

Although *Oreochromis niloticus* DDX43 (OnDDX43) has been demonstrated to activate the IFN-β signaling pathway, the specific biological function of human DDX43 remains unclear due to the relatively low amino acid sequence identity (49%) between the *Oreochromis niloticus* and *Homo sapiens* orthologs [17]. It remains uncertain whether human DDX43 can similarly regulate the expression of IFN, thereby modulating HSV-2 replication. To investigate the molecular mechanisms by which DDX43 suppresses HSV-2 replication, we firstly conducted the temporal analysis of transcriptome of HeLa cells following HSV-2 infection at 0, 2, 4, 6, 8 and 16 hpi. Using fuzzy c-means clustering, 214 pathways were identified and clustered into 8 discrete expression clusters to illustrate the relative expression changes in genes in HeLa cells infected with HSV-2. As shown in Figure 7A, the top 3 pathways enriched in each cluster in the DAVID database were listed next to the clusters. The key pathways highlighted in red are considered to be continuously upregulated during HSV-2 infection, while those highlighted in blue are considered to be continuously downregulated. Considering the antiviral function of DDX43, the three continuously upregulated pathways were selected as the targets for further investigation. It is noteworthy that all three continuously upregulated pathways are closely associated with the IFN-related signaling pathway. Following the transfection of HEK 293T and Vero cells with either pcDNA3.1 (+) or a plasmid expressing DDX43, along with IFN-β-Luc promoter reporter plasmid p125-Luc and internal control plasmid phRL-TK, the dual luciferase report (DLR) assay revealed that overexpression of DDX43 promoted the activation of the IFN-β promoter in 293T cells (Figure 7B). However, due to a spontaneous genetic deletion, the Vero cell line is deficient in the production of IFN-α and IFN-β, and thus overexpression of DDX43 did not enhance the activation of the IFN-β promoter in these cells (Figure 7C).

In viral infections, the IRF-3 mediated signaling pathway is widely recognized for its pivotal role in inducing the expression of type I interferon [25,26]. To identify the potential mechanism by which DDX43 activates IFN-β promoter, the plasmid expressing RIG-I, MAVS, TBK-1, IRF-3/5D or cGAS/STING, which are inducers of IFN-β in the IRF-3 mediated signaling pathway, was transfected into HEK 293T cells together with p125-Luc, phRLTK, plasmid expressing DDX43, or empty vector. As shown in Figure 7D–H, overexpression of RIG-I (Figure 7D), MAVS (Figure 7E), TBK-1 (Figure 7F), IRF-3/5D (Figure 7G) or cGAS/STING (Figure 7H) directly induced the activation of IFN-β promoter. In the presence of DDX43, the activation of IFN promoter by the above components was enhanced, showing that DDX43 promotes RIG-I, MAVS, TBK1, IRF3/5D or cGAS/STING induced IFN-β promoter activation. We further investigated the effect of DDX43 on IFN-β induction. However, DDX43 did not enhance the production of IFN-β at the mRNA level (Figure 7I). Taken together, our data collectively demonstrate that human DDX43 suppresses viral replication through an interferon-independent mechanism and is likely to engage in synergistic interactions with components of the IRF-3-mediated signaling pathway via an undefined mechanism.

## 4. Discussion

Following viral infection, host cells initiate specific defensive responses, often leading to dysregulated gene expressions that characterize viral pathogenesis. Currently, few studies have investigated the differences in HSV-2 replication cross distinct cell lines [12], and the underlying mechanisms responsible for the substantial variability in replication efficiency among these cell types remain poorly understood. In this study, we observed that the yield of progeny viruses in HeLa cells infected with HSV-2 at the same multiplicity of infection (MOI) was significantly lower than that in ARPE-19 cells. Additionally, the size of plaques and syncytia in HeLa cells was notably smaller, and the number of syncytia was significantly reduced compared to ARPE-19 cells. This phenomenon is likely due to differences in the intrinsic regulatory mechanisms within distinct host cells. RNA sequencing was employed on HeLa and ARPE-19 cells at various time points post HSV-2 infection, followed by transcriptomics analysis, to investigate the dynamic changes in cellular components of HeLa and ARPE-19 cells during HSV-2 infection. In the (O)PLS-DA analysis, 25 host genes with the most significant differences in expression were identified. Among these, DDX43 and SORCS3 exhibited the greatest changes in expression, and, to date, no studies have linked them to HSV-1/2 infection. A previous study demonstrated that DDX43 recruits TRIF or IPS-1 as an adaptor to activate the IFN-β pathway in Nile tilapia (*Oreochromis niloticus*) [14]. In this study, we focus on investigating the antiviral function of DDX43 to validate the reliability of the omics strategy and data. It is worthy noting that both SORCS3 and DDX43 are of interest to us, with SORCS3 being investigated in a separate ongoing project.

The DExD/H-box (DDX) helicase family shares a conserved catalytic core, with members exhibiting complex roles in viral infections. While some DDXs have been shown to possess antiviral activities, others have been implicated in facilitating viral infection. Notably, several DDXs exhibit dual functions, both inhibiting and promoting viral replication. For instance, DDX3 has been reported to facilitate viral replication across a wide range of pathogens [27,28,29,30,31,32,33]. Additionally, DDX3 has been shown to exhibit antiviral roles by stimulating interferon production [34] and inhibiting HIV-1 replication [14]. DDX5 has been found to promote viral replication [35,36,37,38,39] and also been shown to inhibit the replication of two DNA viruses, HBV [40] and myxoma virus (MYXV) [41]. Similarly, DDX56 acts as a positive regulator for the replication of several viruses [42,43,44], but serves as a negative regulator for the replication of PRV [15] and Chikungunya virus [16]. Together, these findings suggest that specific members of the DDX family play distinct functional roles during infections caused by different viruses. As a member of the DDX family, the role of DDX43 in viral infections has not been reported. We speculated that the divergent alterations in DDX43 expression levels observed in HSV-2-infected HeLa and ARPE-19 cells may contribute significantly to the difference in progeny virus yield between these two cell lines. DDX43 expression was reduced in HSV-2-infected ARPE-19 cells, which appeared to facilitate the production of HSV-2 progeny viruses. In contrast, the increased expression of DDX43 in HSV-2-infected HeLa cells seemed to inhibit HSV-2 progeny virus production. Further wet experiments confirmed this hypothesis. While overexpression of DDX43 in HeLa cells led to a decrease in the yield of HSV-2 progeny viruses, knockdown of endogenous DDX43 in HeLa cells resulted in an increase in progeny virus yield. These results demonstrated that DDX43 functions as a host regulator to inhibit HSV-2 replication. Our investigation, however, has been limited to exploring the role of DDX43 during HSV-2 infection. Given that various members of the DDX family (including, but not limited to, DDX3, DDX5, and DDX56) exhibit distinct regulatory effects on different viral infections, further studies are warranted to determine whether DDX43 exerts a proviral or antiviral effect on other viruses.

The identification of DDX43 as a host regulator that inhibits HSV-2 replication enhances our understanding of the complex host–pathogen interactions underlying viral pathogenesis, while also offering a promising novel therapeutic target for antiviral intervention. Nevertheless, this study has certain limitations. For instance, in investigating virus–host interactions, single-omics analyses can provide molecular insights at a specific level but often fail to fully capture the complexity of the dynamic regulatory networks between viruses and hosts. Further research should integrate molecular data across various levels, including genomics, transcriptomics, proteomics, and metabolomics to enable a systematic dissection of the spatiotemporal dynamics of virus–host interactions. Such an integrative approach would provide a powerful framework for elucidating viral pathogenesis, characterizing host immune responses, and identifying potential therapeutic targets. Nevertheless, the mechanism by which DDX43 suppresses HSV-2 remains to be further investigated. Given that HSV-2 replication is regulated at multiple stages, including viral attachment, entry, immediate-early gene expression, early gene expression, DNA replication, late gene expression, virion assembly, and egress. Elucidating the underlying mechanism could provide valuable insights for identifying potential therapeutic targets. Based on the observed reductions in gene copy numbers of the immediate-early protein ICP0 and the late protein gB, along with the downregulation of protein expression levels for the major capsid protein ICP5, gB, and gD, it is highly likely that DDX43 exerts its repressive effect at the immediate-early gene expression stage. Further investigations will be conducted to elucidate the underlying molecular mechanisms.

The innate immune system serves as the host’s primary defense against pathogens, operating through rapid detection of pathogen-associated molecular patterns and subsequent induction of effector molecules such as IFNs. IFNs establish a broad-spectrum antiviral state by activating downstream signaling cascades while coordinating adaptive immune responses. Although *Oreochromis niloticus* DDX43 has been shown to activate the IFN-β signaling pathway, our results suggest that human DDX43 may have a distinct functional role on IFN production, as evidenced by the relatively low amino acid sequence identity (49%) between the *O. niloticus* and *H. sapiens* orthologs. In our study, DDX43 was found to potentiate the activation of the IFN-β promoter induced by RIG-I, MAVS, TBK1, IRF3/5D, or the cGAS/STING pathway, yet it did not directly elevate the mRNA expression levels of IFN-β (Figure 7). Our findings suggested that human DDX43 suppresses viral replication through an interferon-independent mechanism and is likely to engage in synergistic interactions with components of the IRF-3-mediated signaling pathway, thereby enhancing IFN-β promoter activity via an as-yet undefined mechanism. However, given that multiple pathways influence IFN production, it cannot be ruled out that human DDX43 may activate IFN-β expression through other mechanisms. Beyond the scope of the present study, additional research will investigate other specific mechanisms through which DDX43 inhibits HSV-2 replication.

## Data Availability

Data are contained within the article.

## Appendix A

**Table A1:** The primers used in PCR and qPCR.

Protein	Primers (5′ → 3′)		Purpose
DDX43	Forward	AGCAGGGGTTAGGGAGTTGT	CDS amplification
	Reverse	CCATCTTGAGAGAGGCATCC	
DDX43	Forward	GGTCCTGAGGGATATAGTGTCG	qPCR
	Reverse	CGATTACCGCGCCAACAAAG	
gB	Forward	CTCGCCGAGCTGTACGT	qPCR
	Reverse	CGGGCGTGGCATTCC	
ICP0	Forward	GTGCATGAAGACCTGGATTCC	qPCR
	Reverse	GGTCACGCCCACTATCAGGTA	
GAPDH	Forward	GGGAAGCTCACTGGCATGG	qPCR
	Reverse	TTACTCCTTGGAGGCCATGT	

**Table A2:** The shRNA sequences employed for the construction of DDX43 knockdown (KD) HeLa cell lines.

shRNAs	Sequences (5′ → 3′)	
shRNA-1	Forward	UUGAUAGAUUGGGAUCAAATT
	Reverse	UUUGAUCCCAAUCUAUCAATT
shRNA-2	Forward	GAGAAUGUUGCAAAUAUUCTT
	Reverse	GAAUAUUUGCAACAUUCUCTT
shRNA-3	Forward	UUGGAUCUAGUUGCUGUAATT
	Reverse	UUACAGCAACUAGAUCCAATT
shRNA-NC	Forward	UUCUCCGAACGUGUCACGUTT
	Reverse	ACGUGACACGUUCGGAGAATT
